# The predictive value of anti-Müllerian hormone on embryo quality in PGT-A cycles analysis

**DOI:** 10.3389/fendo.2025.1645593

**Published:** 2025-12-10

**Authors:** Mengxia Ni, Haoting Zhang, Jian Sun, Gengchao Zhu, Shiyu Xing, Aiyan Zheng, Yan Pu, Guizhi Liao, Jie Ding, Qingxia Meng, Hong Li, Jia Fei, Jian Ou

**Affiliations:** 1 Center for Reproduction and Genetics, The affiliated Suzhou Hospital of Nanjing Medical University, Suzhou Municipal Hospital, Suzhou, Jiangsu, China; 2 Research and Development Department, Peking Jabrehoo Medical Technology Company Limited, Beijing, China; 3 Department of Laboratory Medicine, The affiliated Suzhou Hospital of Nanjing Medical University, Suzhou Municipal Hospital, Suzhou, Jiangsu, China

**Keywords:** anti-Müllerian hormone, preimplantation genetic aneuploidy test, female age, body mass index, embryo quality

## Abstract

**Objective:**

Although anti-Müllerian hormone (AMH) is a well-established predictor of oocyte number in assisted reproduction (ART) applications, its potential to reflect embryo quality in ART cycles remains uncertain. This study explored the association between AMH levels and embryo quality, using aneuploidy rates from preimplantation genetic testing for aneuploidy (PGT-A) as an objective indicator of embryo quality.

**Methods:**

A retrospective analysis was performed on patients (excluding those with male factor infertility) who underwent PGT-A at the Reproduction and Genetics Center of Suzhou Municipal Hospital (2018-2022). Patients were stratified by age (<35, 35-37, ≥38 years), body mass index (BMI) (<18.5, 18.5-25, ≥25 kg/m²), number of viable embryos (1-2, 3-4, ≥5), and AMH levels. Group comparisons used independent samples t-test, and correlations with embryo quality were analyzed via logistic regression.

**Results:**

In 542 PGT-A cycles, AMH levels, euploidy and mosaicism rates negatively correlated with age, while, aneuploidy rates were positively correlated with age. BMI group showed AMH levels shows no significant differences between the groups. However, there were significant differences in euploid, aneuploidy rates among the BMI groups(≥25 kg/m²vs <18.5 kg/m²; 18.5–25 kg/m²vs <18.5 kg/m²). Both AMH levels and euploidy rates were positively correlated with the number of viable embryos, while the aneuploidy rate was correlated negatively. The>1.68 ng/mL AMH group had significantly higher euploidy and lower aneuploidy rates. AMH levels negatively correlated with aneuploidy rates.

**Conclusion:**

AMH levels serve as a valuable predictor of embryo quality, with the >1.68 ng/mL group showing better quality than the ≤1.68 ng/mL group. AMH levels strongly correlate with female age and the number of biopsy embryos but not BMI. Embryo quality demonstrated a significant decline with increasing female age and a marked improvement with a higher number of biopsy embryos and higher AMH levels. BMI did not linear correlation with embryo quality, thus cannot be considered an independent predictor of embryo quality.

## Introduction

AMH belongs to the β superfamily of transforming growth factor and is a homodimeric glycoprotein (1), which is synthesized by granulosa cells in preantral and small follicles in the ovaries. It influences primordial follicles by regulating the autophagy process through synergistic interactions with other substances (2–4). Follicle-Stimulating Hormone (FSH) is a key hormone that drives the growth and maturation of ovarian follicles. AMH limits the role of FSH in small growing follicles through two core mechanisms: downregulating FSH receptor expression to reduce follicular sensitivity to FSH and inhibiting FSH-mediated follicular growth signaling pathways (5). In summary, AMH is highly concentrated on early-stage follicles: it does not act directly on follicles that have entered the mature stage, but specifically targets small growing follicles that have not yet developed a strong dependence on FSH. By regulating the growth rhythm of these “reserve follicles”, AMH lays the foundation for the long-term stability of ovarian function, and it has been widely used in the clinic to assess ovarian function.

In PGT-A, we need to consider and overcome a number of factors that may have an impact on the outcome, including the patient’s ovarian responsiveness, the ovulation regimen, the initiating dose of gonadotropins (Gn), endometrial tolerance, and the quality of the eggs and embryos (6). It has been shown that the direct factor associated with implantation is the quality of eggs and embryos, while the number of eggs acquired and the number of mature eggs directly affects the number of embryos and the number of good quality embryos (7, 8). Embryonic aneuploidy can be used as an objective qualitative factor to reflect the quality of eggs and embryos, while AMH, as an optimal indicator for assessing ovarian function, is controversial as to whether or not there is a correlation between AMH, as an indicator of ovarian function, and the quality of oocytes and embryos (9–11).

Therefore, in this study, we analyzed 542 PGT-A cycles in our center to find out the relationship between AMH level and the number of embryos obtained and embryo quality, and at the same time statistically analyzed its relationship with age and BMI level, in order to further evaluate whether AMH can be used as a predictor of embryo quality, and to clarify its correlation which can provide some new bases for clinical diagnosis and treatment.

## Materials and methods

### Study objects

**Figure 1:**
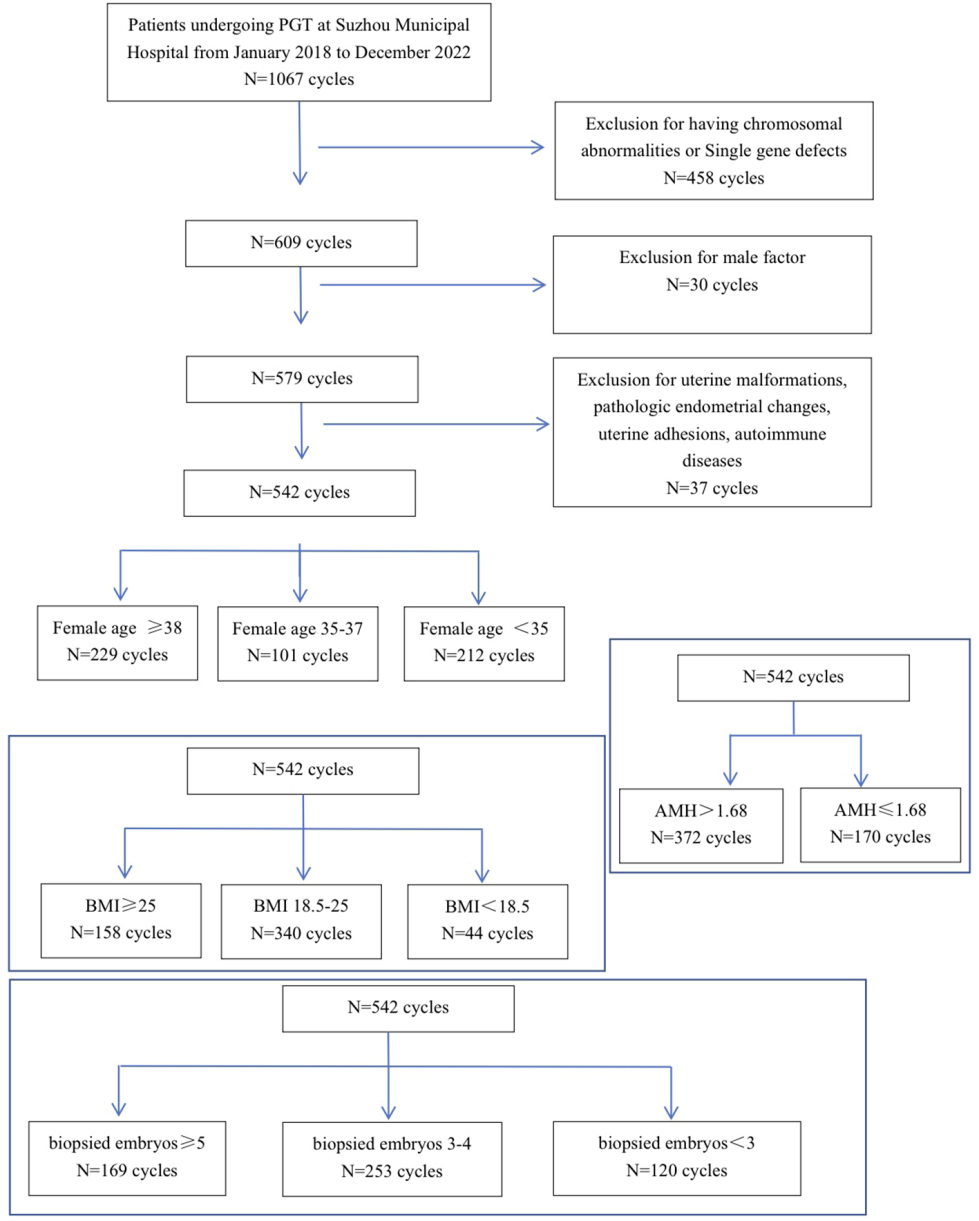
Flowchart of patient screening and stratification of female age, BMI, number of viable embryos, and AMH levels.

Patients (without male factor) who underwent PGT-A at the Center for Reproduction and Genetics of Suzhou Municipal Hospital from 2018–2022 were collected for retrospective analysis, and a total of 2,171 embryos from 542 cycles were included in the study. Exclusion criteria: known uterine malformations, pathologic endometrial changes, uterine adhesions, autoimmune diseases, etc. Female age, BMI, AMH, and number of viable embryos were summarized and counted(**Figure 1**). The study subjects signed the relevant informed consent. The research was approved by the Ethics Committee of Suzhou Municipal Hospital before it was conducted.

### Anti-Müllerian hormone testing

Before the start of the ART cycle, peripheral blood was collected from female patients on the 2nd-3rd day of menstruation for serum AMH testing. Serum AMH levels are measured using a second-generation enzyme-linked immunosorbent assay (Immunotech, Beckman Coulter Inc., USA). Samples can be accurately measured within the analytical range of the lower limit of detection and the highest calibration value (approximately 0.02–24 ng/mL [0.14–171 pmol/L]).

### Embryonic aneuploidy detection

The IVF cycle used a long protocol or a GnRH (gonadotropin-releasing hormone) antagonist protocol to control ovarian hyperstimulation. Oocytes were collected after hCG injection 34-35hours, and fertilized by single sperm intracytoplasmic injection (ICSI). All embryos were incubated with laser technique on day 3 and then placed in fresh medium until day 5 or 6 after which they could be considered for biopsy if they had developed into complete blastomeres. Whole genome amplification (REPLI-g Single Cell WGA Kit, Qiagen, Germany)was performed on all biopsy samples and then libraries were constructed. After isothermal amplification and enrichment, sequencing was performed using a PGM sequencer (ABI Ion Torrent PGM, USA). Sequencing results were interpreted using Ion Reporter software, and chromosome analysis was performed by analyzing the data through the interface or the background linux interface to determine the aneuploidy of the embryos.

### Group analysis

Participants were categorized based on age (<35, 35-37, ≥38), body mass index (BMI) (<18.5 kg/m², 18.5–25 kg/m², ≥25 kg/m²), the number of embryos available for biopsy (1-2, 3-4, ≥5), and AMH levels (≤1.68 ng/ml, >1.68 ng/ml).

### Statistical methods

Euploidy rate, defined as the number of euploid blastocysts divided by the number of biopsied blastocysts per cycle. Aneuploidy rate, defined as the number of aneuploidy blastocysts per cycle divided by the number of biopsy blastocysts. Statistical analysis was performed using SPSS 19.0. Normally distributed quantitative data were expressed as mean ± standard deviation (Mean ± SD). Independent samples t-test was used for comparison between groups; P<0.05 was considered as statistically significant difference. Logistic regression was used to evaluate the association between aneuploidy rate and female age, BMI, number of viable embryos, and AMH levels.

## Results

### Comparison of AMH and embryo quality analysis results stratified by age

**Figure 2:**
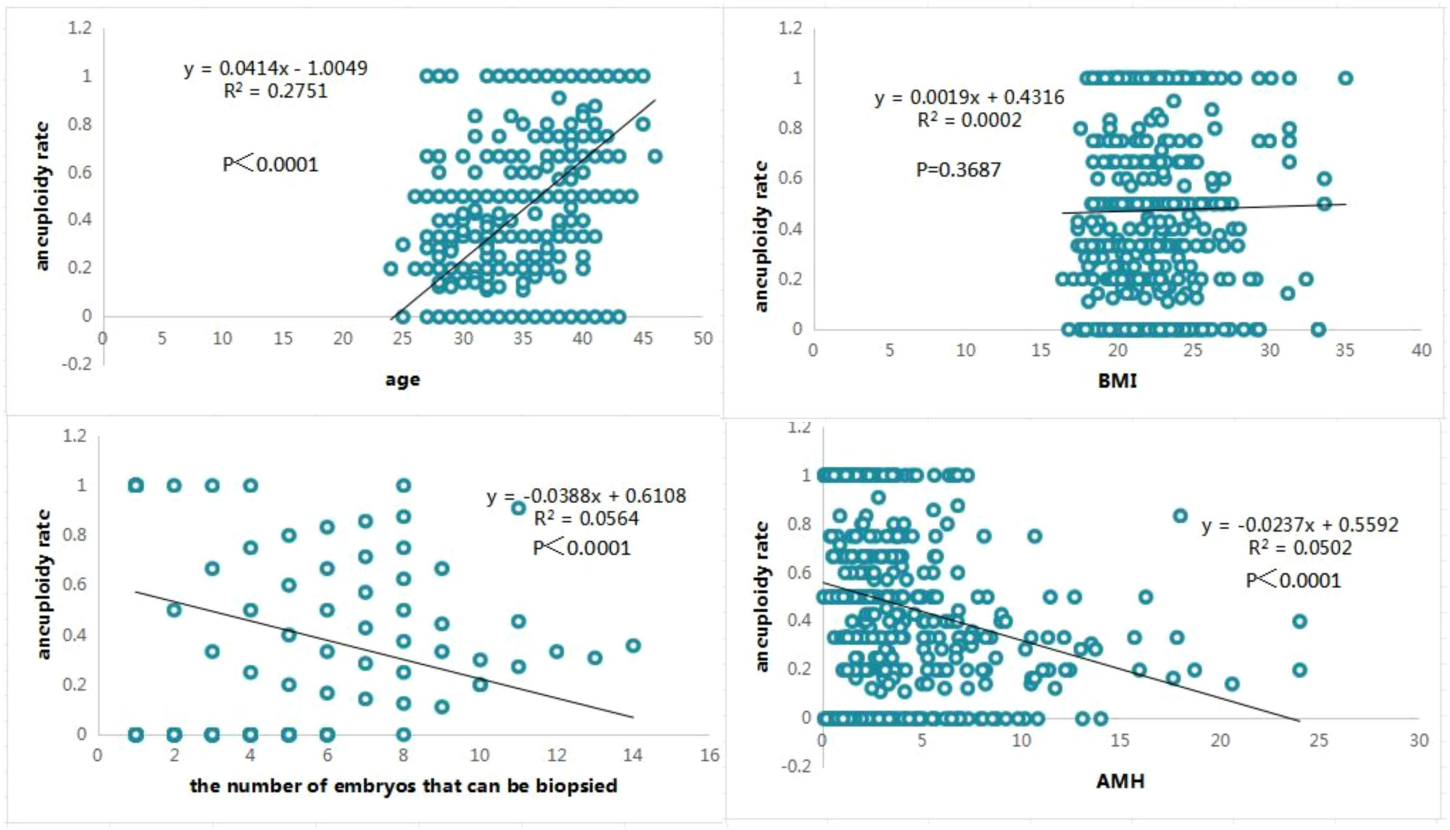
The logistic regression analysis for aneuploidy rate (age, BMI, the number of embryos that can be biopsied and AMH).

In 542 PGT-A cycles, stratified by age(<35, 35-37, ≥38), the AMH level(5.31 ± 4.3 vs. 3.12 ± 2.79vs. 2.22 ± 1.84), the euploidy rate of embryos(0.54 ± 0.3 vs. 0.44 ± 0.32vs. 0.25 ± 0.33), and the mosaicism rate(0.18 ± 0.23 vs. 0.13 ± 0.21vs. 0.07 ± 0.19)exhibited a negative correlation with age, whereas the aneuploidy rate demonstrated a positive correlation with age (0.27 ± 0.27 vs. 0.43 ± 0.33vs. 0.67 ± 0.36) (**Table 1**). The t-test showed that there were significant differences between all two groups of analyses. The logistic regression analysis showed the strongest positive correlation between age and percentage of aneuploid embryos (P<0.0001, R^2^ = 0.2751) (**Figure 2**).

**Table 1:** AMH and embryo quality analysis results stratified by age. .

AMH and embryo quality	age≥38 (n=229)	age 35-37 (n=101)	age<35 (n=212)	P
AMH	2.22 ± 1.84^a,c^	3.12 ± 2.79^a,b^	5.31 ± 4.3^b,c^	^a^*p* = 0.001,^b^*p*<0.0005 ^c^*p*<0.0005
euploidy rate	0.25 ± 0.33^a,c^	0.44 ± 0.32^a,b^	0.54 ± 0.3^b,c^	^a^*p* = 0.001,^b^*p*<0.0005 ^c^*p*<0.007
aneuploidy rate	0.67 ± 0.36^a,c^	0.43 ± 0.33^a,b^	0.27 ± 0.27^b,c^	^a^*p*<0.0005,^b^*p*<0.0005 ^c^*p*<0.0005
mosaicism rate	0.07 ± 0.19^a,c^	0.13 ± 0.21^a,b^	0.18 ± 0.23^b,c^	^a^*p*<0.014,^b^*p*<0.0005 ^c^*p*<0.0005

Values presented as mean ± SD.

^a,b,c^Statistical significance (P < 0.05) among groups in the independent samples t-test analysis.

### Comparison of AMH and embryo quality analysis results stratified by BMI

BMI(<18.5 kg/m², 18.5–25 kg/m², ≥25 kg/m²), AMH levels(4.3 ± 3.55 vs. 4.84 ± 3.37vs. 3.77 ± 2.54) shows no significant differences between the groups. However, there were significant differences in euploid(p=0.002, 0.24 ± 0.23 vs. 0.46 ± 0.34; p<0.0005 0.27 ± 0.22 vs. 0.46 ± 0.34), aneuploidy rates(p=0.002, 0.67 ± 0.26 vs. 0.44 ± 0.37; p<0.0005 0.63 ± 0.27 vs. 0.44 ± 0.37) among the BMI groups(BMI≥25 kg/m²vs <18.5 kg/m²; 18.5–25 kg/m²vs <18.5 kg/m²) (**Table 2**). Nevertheless, the correlation was not observed between BMI and aneuploid embryos from the logistic regression analysis (**Figure 2**).

**Table 2:** AMH and embryo quality analysis results stratified by BMI.

AMH and embryo quality	BMI≥25 (n=158)	BMI18.5-25 (n=340)	BMI<18.5 (n=44)	P
AMH	3.77 ± 2.54	4.84 ± 3.37	4.3 ± 3.55	
euploidy rate	0.24 ± 0.23^a^	0.27 ± 0.22^b^	0.46 ± 0.34^a,b^	^a^*p* = 0.002,^b^*p*<0.0005
aneuploidy rate	0.67 ± 0.26^a^	0.63 ± 0.27^b^	0.44 ± 0.37^a,b^	^a^*p* = 0.002,^b^*p*<0.0005
mosaicism rate	0.08 ± 0.14	0.1 ± 0.18	0.1 ± 0.13	

Values presented as mean ± SD.

^a,b^Statistical significance (P < 0.05) among groups in the independent samples t-test analysis.

### Comparison of AMH and embryo quality analysis results stratified by the number of embryos that can be biopsied

When stratified by the number of embryos available for biopsy (1-2, 3-4, ≥5), both the AMH level(2.04 ± 2.01 vs. 3.37 ± 2.65vs. 5.75 ± 4.4) and the euploidy rate of embryos(0.29 ± 0.4 vs. 0.41 ± 0.3vs. 0.53 ± 0.24) were positively correlated with the number of biopsied embryos, whereas the aneuploidy rate(0.59 ± 0.44 vs. 0.46 ± 0.31vs. 0.34 ± 0.25) was negatively correlated with the number of biopsied embryos (**Table 3**). The t-test showed that there were significant differences between all two groups of analyses except the mosaicism rate group. The logistic regression analysis showed significant negative correlation between the number of biopsied embryos and percentage of aneuploid embryos (P<0.0001), but this association is not particularly robust (R^2^ = 0.0564) (**Figure 2**).

**Table 3:** AMH and embryo quality analysis results stratified by the number of embryos that can be biopsied.

AMH and embryo quality	biopsied embryos≥5 (n=169)	biopsied embryos3-4 (n=163)	biopsied embryos<3 (n=120)	P
AMH	5.75 ± 4.4^a,c^	3.37 ± 2.65^a,b^	2.04 ± 2.01^b,c^	^a^*p*<0.0005,^b^*p*<0.0005 ^c^*p*<0.0005
euploidy rate	0.53 ± 0.24^a,c^	0.41 ± 0.3^a,b^	0.29 ± 0.4^b,c^	^a^*p*<0.0005,^b^*p* = 0.002 ^c^*p*<0.0005
aneuploidy rate	0.34 ± 0.25^a,c^	0.46 ± 0.31^a,b^	0.59 ± 0.44^b,c^	^a^*p* = 0.001,^b^*p* = 0.001 ^c^*p*<0.0005
mosaicism rate	0.13 ± 0.14	0.13 ± 0.18^a^	0.12 ± 0.28^a^	^a^*p* = 0.001

Values presented as mean ± SD.

^a,b,c^Statistical significance (P < 0.05) among groups in the independent samples t-test analysis.

### Comparison of embryo quality analysis results stratified by AMH

Stratification by AMH level (≤1.68 ng/ml, >1.68 ng/ml) revealed the x̅ ± SD for each parameter; the euploidy rate of embryos was significantly higher in the >1.68 ng/ml group compared to the ≤1.68 ng/ml group (P = 0.017, 0.35 ± 0.38 vs. 0.42 ± 0.32)and aneuploidy rate of embryos was significantly lower(P = 0.025, 0.53 ± 0.40 vs. 0.45 ± 0.35). (**Table 4**). The logistic regression analysis showed significant negative correlation between AMH level and percentage of aneuploid embryos (P<0.0001), but the strength of correlation remaining moderate (R^2^ = 0.0502) (**Figure 2**).

**Table 4:** Embryo quality analysis results stratified by AMH.

Embryo quality	AMH>1.68(n=372)	AMH ≤ 1.68 (n=170)	P
euploidy rate	0.42 ± 0.32	0.35 ± 0.38	*p* = 0.017
aneuploidy rate	0.45 ± 0.35	0.53 ± 0.40	*p* = 0.025
mosaicism rate	0.13 ± 0.14	0.13 ± 0.27	

Values presented as mean ± SD.

Statistical significance (P < 0.05) among groups in the independent samples t-test analysis.

## Discussion

Currently, assisted human reproduction technology has significantly advanced; however, enhancing the clinical pregnancy rate remains a subject warranting further investigation. AMH, a peptide mainly secreted by the gonads, plays a crucial role in human reproduction. It operates independently of the menstrual cycle and exhibits autocrine, endocrine, and paracrine functions within the reproductive system. AMH’s significance in the field of assisted reproduction stems from its correlation with a woman’s age and the number of antral follicles, making it a valuable marker for assessing ovarian reserve and ovarian response during ovulation induction. Nonetheless, its predictive value for embryo quality and pregnancy outcomes remains inconclusive (12–16).

In this study, by retrospectively analyzing 542 patients in PGT-A cycles, categorized by age, BMI, number of biopsied embryos, and AMH levels, we found a significant correlation between female age and embryo quality. The relationship between female age and embryo quality can be attributed to the impact of oocyte quality on embryo quality. It is well-documented that age-related declines in ovarian reserve and oocyte quality can hinder the development of fertilized eggs, leading to a higher rate of aneuploidy (17), which is align with the findings of this study. In addition, we found that the mosaicism rate of embryos decreased significantly in the older age group, as the reports said mosaicism were more prevalent among younger patients (18, 19), which may indicate that with age, the embryo’s ability to self-repair chromosomal abnormalities in the early cleavage decreases, which corresponds to a decrease in mosaicism.

Prior studies have shown that BMI has an inverse association with AMH levels in patients with polycystic ovarian syndrome (PCOS) (20, 21), but our data showed no difference between AMH and BMI in PGT-A cycles. Grouping by BMI revealed no correlation with AMH values but with embryo quality, the euploid and aneuploidy rates of the embryos show statistically significant differences (≥25 kg/m²vs <18.5 kg/m²; 18.5–25 kg/m²vs <18.5 kg/m²). Our previous data (unpublished) have shown that weight loss in some PGT patients leads to a higher number of euploid embryos in subsequent PGT cycles. This suggests that individual weight regulation may improve embryo quality. But other studies investigated the number and percentage of euploid, aneuploid and mosaic embryos did not vary according to BMI (22, 23), further studies involving larger cohorts are necessary. However, high BMI also can lead to an increased risk of miscarriage after euploid embryo transfer (24), these women trying to become pregnant should be advised weight reduction before pregnancy.

Some studies have shown that the pregnancy rate increases with the increase of the number of eggs while acquired eggs ≤ 15, the pregnancy rate does not change significantly between 15 and 20 eggs acquired, and the pregnancy rate decreases with the increase of the number of eggs acquired when the number of eggs acquired is > 20 (25), which shows that the number of eggs acquired is not the more the better, but the quality of the eggs and the quality of the embryos that are formed afterward is more important. At this stage, all the embryos available for biopsy in our center are relatively high-quality 5-6-day blastocysts, which to some extent reflects the quality of the embryos. At this stage, AMH serves as an excellent indicator of ovarian reserve, with numerous studies establishing models to predict oocyte yield based on AMH levels (26), and our statistics grouped according to the number of embryos biopsied demonstrated a strong linear correlation with AMH levels, which can also be considered for further prediction of the number of embryos biopsied. In addition, our results show that the number of embryos biopsied is positively correlated with the euploidy rate of embryos and negatively correlated with the aneuploidy rate of embryos, which can be a good predictor of PGT-A outcome.

Grouping by AMH values showed significant difference in embryo quality between the low (≤1.68 ng/mL) and the high AMH group (>1.68 ng/mL). We selected 1.68 ng/mL (12 pmol/L) as the AMH cutoff based on Arce et al. (27), who demonstrated its superiority in predicting both low oocyte yield and cumulative live birth rate—outcomes directly relevant to PGT-A success—compared to thresholds focused solely on poor ovarian response diagnosis (28, 29). Given the predictive value of AMH levels for embryo quality, they can be used clinically to anticipate oocyte and embryo conditions and guide ovulation induction protocols. Previous papers have also been controversial on whether AMH is correlated with aneuploidy rates in embryos. For example, Pipari’s report concluded that AMH is not correlated with aneuploidy rates (30), but the correlation between age and aneuploidy rates in synchronous statistics did not reach a very significant difference, which is also inconsistent with the statistical results of other papers (31, 32).Although female age is more favorable than AMH in predicting aneuploidy in embryos, younger women with lower ovarian reserve appeared to have better outcomes than older women with good ovarian reserve, higher AMH values may partially mitigate the age-related decline in live birth rates in older women (33). When comparing AMH and female age, AMH is a superior predictor of oocyte yield, miscarriage and live birth compared to female age (34, 35).

In conclusion, combining the results of the present study and previous studies, serum AMH levels were found to be strongly associated with female age and the number of embryos biopsied. AMH levels also serve as a valuable predictor of embryo quality. Therefore, it can be used as a reference indicator to predict embryo quality when factors such as age are not effective. In our follow-up work, we can also try to establish a relevant model to predict embryo quality by AMH value. Our data can help individualized counseling for patients preparing for PGT-A. Since this study was based on a limited number of cases and data from a single center, these findings need to be confirmed by a larger cohort study.

## Data Availability

The original contributions presented in the study are included in the article/supplementary material. Further inquiries can be directed to the corresponding author.
